# Genome Sequences of Gordonia rubripertincta Bacteriophages Jellybones and NHagos

**DOI:** 10.1128/MRA.00935-20

**Published:** 2020-10-01

**Authors:** D'Andrew L. Harrington, Jennifer L. Stevens, Mia J. Johnson, Samantha J. Pochiro, Michelle M. Moriarty, Miriam E. Robertson, Andy Sanchez, Orin G. Whitby, Kendra W. Kimberley, Chelsey C. McKenna, James R. Theoret, Erin J. Windsor, Earl J. Yoon

**Affiliations:** a Department of Biology, College of Southern Nevada, Las Vegas, Nevada, USA; Portland State University

## Abstract

Jellybones and NHagos are bacteriophages that were identified in the host bacterium Gordonia rubripertincta NRRL B-16540. Jellybones has a direct terminal repeat and was assigned to the CS2 subcluster with a length of 77,514 bp. NHagos is circularly permuted and was assigned to the DR cluster with a length of 59,580 bp.

## ANNOUNCEMENT

**FIG 1:**
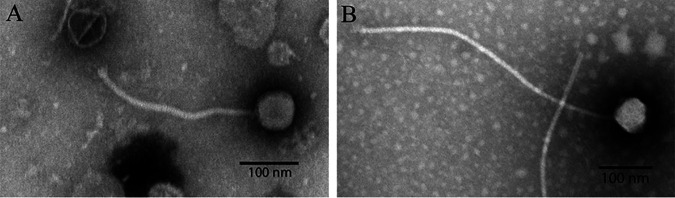
Transmission electron micrographs of bacteriophages NHagos (A) and Jellybones (B) reveal morphologies characteristic of the *Siphoviridae* family after negative staining with 1% phosphotungstic acid.

To date, there have been only 36 bacteriophages sequenced from the actinobacterial host Gordonia rubripertincta NRRL B-16540 (1). These bacteriophages are classified into eight clusters in the PhagesDB database (1). Gordonia rubripertincta, a Gram-positive, opportunistic pathogen, affects hospitalized patients with catheter-related infections (2). Both bacteriophages were isolated as part of the Howard Hughes Medical Institute (HHMI) Science Education Alliance-Phage Hunters Advancing Genomics and Evolutionary Science (SEA-PHAGES) programs at the College of Southern Nevada (3, 4). Jellybones was collected from soil at a bird-viewing preserve in Henderson, Nevada (GPS coordinates, 36.076304N, 115.002681W). Jellybones produces lytic plaques ranging from 0.5 mm to 1 mm in size with distinct borders. NHagos was collected from soil at a flamingo habitat in Las Vegas, Nevada (GPS coordinates, 36.11659N, 115.17107W). NHagos produces 0.5-mm lytic plaques. An enriched isolation method was used. Environmental samples were incubated with 500 μl of bacteria and shaken (250 rpm, overnight) at 30°C, followed by centrifugation and filter sterilization (0.22 μm) of the supernatant according to the HHMI SEA-PHAGES Phage Discovery Guide (5). Bacteriophages were purified via three rounds of plaque assays. Purified bacteriophage was prepared with 1% phosphotungstic acid on a copper grid and sent to Brigham Young University for imaging (5). Transmission electron microscopy revealed the *Siphoviridae* morphology for both bacteriophages (Fig. 1A and B). DNA was extracted from 2 ml of supernatant using the Promega Wizard DNA clean-up system (catalog number A7280) according to the manufacturer’s instructions. The NHagos and Jellybones genomes were sequenced by the Pittsburgh Bacteriophage Institute. Sequencing libraries were created from extracted genomic DNA using New England Biolabs Ultra II kits according to the manufacturer’s instructions. The libraries were sequenced with an Illumina MiSeq instrument to provide 150-bp single-end reads with 1,592-fold coverage for Jellybones and 1,672-fold coverage for NHagos. The reads (856,721 reads for Jellybones and 68,340 reads for NHagos) were quality controlled and assembled using Newbler v2.9 with default settings and analyzed using Consed v29 with default settings to produce a single contig, which was evaluated for accuracy, completeness, and bacteriophage genomic termini (6). Jellybones, with 191-bp terminal repeat genomic termini, has a length of 77,514 bp and a GC content of 59%. NHagos, with circularly permuted genomic termini, has a length of 59,580 bp and a GC content of 68.2%.

Annotation of Jellybones and NHagos was completed using the following programs: DNA Master v5.23.2 (http://cobamide2.bio.pitt.edu/computer.htm), Starterator v1.2 (https://github.com/SEA-PHAGES/starterator), Phamerator (https://phamerator.org) (7), PhagesDB BLAST (phagesdb.org/blast) (1), NCBI BLAST (8), PECAAN (https://pecaan.kbrinsgd.org/), GeneMark v2.5p (9), Glimmer v3.02 (10), ARAGORN v1.1 and v1.2.38 (11), HHpred v3.2.0 (12), tRNAscan-SE v2.0 (13), TMHMM v2.0 (14), and SOSUI v1.11 (15–17).

Bacteriophages were placed into clusters as described previously (3). Jellybones has the third longest genome in the CS2 cluster. One tRNA-Asn was identified within Jellybones. Genes 1 to 8 and 43 to 109 are all reverse. NHagos, like the other DR cluster members, has a similar layout of alternating forward and reverse genes. A frameshift mutation was identified in the tail assembly chaperone proteins of both bacteriophages. Functions were predicted for 33 genes in Jellybones and 34 genes in NHagos.

### Data availability.

The GenBank and SRA accession numbers for Jellybones are MN444874 and SRR11178667, respectively. The GenBank and SRA accession numbers for NHagos are MN369758 and SRR11178666, respectively.

## References

[1] Russell DA, Hatfull GF 2017 PhagesDB: the actinobacteriophage database. Bioinformatics 33:784–786. doi:10.1093/bioinformatics/btw711. 28365761 PMC5860397

[2] Grisold AJ, Roll P, Hoenigl M, Feierl G, Vicenzi-Moser R, Marth E 2007 Isolation of *Gordonia terrae* from a patient with catheter-related bacteraemia. J Med Microbiol 56:1687–1688. doi:10.1099/jmm.0.47388-0. 18033840

[3] Pope WH, Bowman CA, Russell DA, Jacobs-Sera D, Asai DJ, Cresawn SG, Jacobs WR, Hendrix RW, Lawrence JG, Hatfull GF, Science Education Alliance-Phage Hunters Advancing Genomics and Evolutionary Science, Phage Hunters Integrating Research and Education, Mycobacterial Genetics Course. 2015 Whole genome comparison of a large collection of mycobacteriophages reveals a continuum of phage genetic diversity. Elife 4:e06416. doi:10.7554/eLife.06416. 25919952 PMC4408529

[4] Pope WH, Jacobs-Sera D, Russel AR, Cresaw GS, Hatfull GF 2017 SEA-PHAGES bioinformatics guide. Howard Hughes Medical Institute, Chevy Chase, MD.

[5] Poxleitner M, Pope W, Jacobs-Sera D, Sivanathan V, Hatfull G 2018 Phage discovery guide. Howard Hughes Medical Institute, Chevy Chase, MD.

[6] Russell DA 2018 Sequencing, assembling, and finishing complete bacteriophage genomes. Methods Mol Biol 1681:109–125. doi:10.1007/978-1-4939-7343-9_9. 29134591

[7] Cresawn SG, Bogel M, Day N, Jacobs-Sera D, Hendrix RW, Hatfull GF 2011 Phamerator: a bioinformatic tool for comparative bacteriophage genomics. BMC Bioinformatics 12:395. doi:10.1186/1471-2105-12-395. 21991981 PMC3233612

[8] Altschul SF, Gish W, Miller W, Myers EW, Lipman DJ 1990 Basic local alignment search tool. J Mol Biol 215:403–410. doi:10.1016/S0022-2836(05)80360-2. 2231712

[9] Besemer J, Borodovsky M 2005 GeneMark: Web software for gene finding in prokaryotes, eukaryotes and viruses. Nucleic Acids Res 33:W451–W454. doi:10.1093/nar/gki487. 15980510 PMC1160247

[10] Delcher AL, Bratke KA, Powers EC, Salzberg SL 2007 Identifying bacterial genes and endosymbiont DNA with Glimmer. Bioinformatics 23:673–679. doi:10.1093/bioinformatics/btm009. 17237039 PMC2387122

[11] Laslett D, Canback B 2004 ARAGORN, a program to detect tRNA genes and tmRNA genes in nucleotide sequences. Nucleic Acids Res 32:11–16. doi:10.1093/nar/gkh152. 14704338 PMC373265

[12] Remmert M, Biegert A, Hauser A, Söding J 2011 HHblits: lightning-fast iterative protein sequence searching by HMM-HMM alignment. Nat Methods 9:173–175. doi:10.1038/nmeth.1818. 22198341

[13] Lowe TM, Chan PP 2016 tRNAscan-SE on-line: integrating search and contextual analysis of transfer RNA genes. Nucleic Acids Res 44:W54–W57. doi:10.1093/nar/gkw413. 27174935 PMC4987944

[14] Krogh A, Larsson B, Von Heijne G, Sonnhammer EL 2001 Predicting transmembrane protein topology with a hidden Markov model: application to complete genomes. J Mol Biol 305:567–580. doi:10.1006/jmbi.2000.4315. 11152613

[15] Hirokawa T, Boon-Chieng S, Mitaku S 1998 SOSUI: classification and secondary structure prediction system for membrane proteins. Bioinformatics 14:378–379. doi:10.1093/bioinformatics/14.4.378. 9632836

[16] Mitaku S, Hirokawa T 1999 Physicochemical factors for discriminating between soluble and membrane proteins: hydrophobicity of helical segments and protein length. Protein Eng Des Sel 12:953–957. doi:10.1093/protein/12.11.953. 10585500

[17] Mitaku S, Hirokawa T, Tsuji T 2002 Amphiphilicity index of polar amino acids as an aid in the characterization of amino acid preference at membrane-water interfaces. Bioinformatics 18:608–616. doi:10.1093/bioinformatics/18.4.608. 12016058

